# Kinfitr — an open-source tool for reproducible PET modelling: validation and evaluation of test-retest reliability

**DOI:** 10.1186/s13550-020-00664-8

**Published:** 2020-07-08

**Authors:** Jonathan Tjerkaski, Simon Cervenka, Lars Farde, Granville James Matheson

**Affiliations:** Department of Clinical Neuroscience, Centre for Psychiatry Research, Karolinska Institutet & Stockholm Health Care Services, Stockholm County Council, Karolinska University Hospital, SE-171 76 Stockholm, Sweden

**Keywords:** Positron emission tomography, Kinetic modelling, Reproducible research, R

## Abstract

**Background:**

In positron emission tomography (PET) imaging, binding is typically estimated by fitting pharmacokinetic models to the series of measurements of radioactivity in the target tissue following intravenous injection of a radioligand. However, there are multiple different models to choose from and numerous analytical decisions that must be made when modelling PET data. Therefore, it is important that analysis tools be adapted to the specific circumstances, and that analyses be documented in a transparent manner. *Kinfitr*, written in the open-source programming language R, is a tool developed for flexible and reproducible kinetic modelling of PET data, i.e. performing all steps using code which can be publicly shared in analysis notebooks. In this study, we compared outcomes obtained using *kinfitr* with those obtained using PMOD: a widely used commercial tool.

**Results:**

Using previously collected test-retest data obtained with four different radioligands, a total of six different kinetic models were fitted to time-activity curves derived from different brain regions. We observed good correspondence between the two kinetic modelling tools both for binding estimates and for microparameters. Likewise, no substantial differences were observed in the test-retest reliability estimates between the two tools.

**Conclusions:**

In summary, we showed excellent agreement between the open-source R package *kinfitr*, and the widely used commercial application PMOD. We, therefore, conclude that *kinfitr* is a valid and reliable tool for kinetic modelling of PET data.

## Background

Positron emission tomography (PET) is an imaging modality with high sensitivity and specificity for biochemical markers and metabolic processes in vivo [1]. It is an important tool in the study of psychiatric and neurological diseases, as well as for evaluating novel and established pharmacological treatments [2–4]. In PET imaging, study participants receive an intravenous injection of a radioligand, which binds specifically to a target molecule [5]. The concentration of radioligand in a region of interest (ROI) is measured over time to produce a time-activity curve (TAC) [6]. Radioligand binding, and thereby the concentration of the target molecule, can then be estimated using quantitative kinetic models [7, 8], of which there are many.

Importantly, the choice of a certain kinetic modelling approach should be based on several considerations, including the pharmacokinetic properties of the radioligand, the signal-to-noise ratio of the TAC, the availability of arterial blood sampling and the biological research question. Furthermore, there are various other analytical decisions that must be made in conjunction with modelling, such as the selection of statistical weighting schemes, t* values and reasonable parameter bounds for iterative fitting methods. The sheer number of options available for kinetic modelling, in addition to those in prior pre-processing of image data [9] and blood data [10, 11], makes it important that analyses can not only be flexibly adjusted to the circumstances, but also that all steps are carefully documented. In this context, full communication of all analytical steps and decisions, as well as their motivations, may not be practically feasible within the confines of a scientific publication. This issue is common to all fields making extensive use of scientific computing, impeding replication efforts and obscuring potential errors [12]. A recent consensus paper [13] presented guidelines for the content and format of PET study reporting, and which information is considered mandatory, recommended or optional, which aims to standardize the communication of PET analyses. An additional, and more comprehensive approach to this problem, is the adoption of reproducible research practices: this means increasing transparency by exposing the research workflow to the scientific community, through sharing of analysis code and (when possible) data [12, 14, 15]. This allows an independent observer to easily inspect and reproduce ones work, and, if necessary, interrogate the sensitivity of the outcomes to the chosen strategy. Reproducible analysis also has the advantage of automatically documenting the steps taken in the research code itself, rather than in complicated log files. This further benefits the analyst, as modifications can be made to the analysis, or data updated, and the code can simply be rerun, rather than requiring that all steps be taken anew.

Several tools, both commercial and open-source, have been developed to facilitate the analysis of PET data [16–19]. These tools differ in their focus on various levels of analysis such as image reconstruction, image processing or high-throughput quantification. *Kinfitr* is an open-source software package specifically developed for the purpose of performing PET kinetic modelling. It is written in the R programming language [20], which provides access to a rich ecosystem of tools for reproducible research. The overall aims of *kinfitr* are to provide researchers with a high degree of flexibility during modelling as well as to provide the user with the ability to report all the steps taken during this process in a transparent manner [21]. This software package has been used in several scientific publications (e.g. [22–25]); however, it has not yet been formally evaluated against other software. This is an important safeguard for open-source software, as bugs could otherwise go unnoticed (for example, one such study identified a 15-year-old bug in a commonly used neuroimaging tool [26]).

The purpose of this study was to validate *kinfitr* for use in applied studies, by comparing its estimates using real data to those obtained with the widely used commercially available software PMOD [18], which we will use as a reference point for the purposes of this analysis. Making use of previously collected test-retest data for four different radioligands, we evaluated the agreement between these tools, using three different kinetic models each.

## Methods

### Data and study participants

This study was performed using data from four previous studies carried out at the Centre for Psychiatry Research, Department of Clinical Neuroscience, Karolinska Institutet, Stockholm, Sweden. In all studies, the data collection was approved by the Regional Ethics and Radiation Safety Committee of the Karolinska Hospital, and all subjects had provided written informed consent prior to their participation. All participants were young (aged 20–35 years), healthy individuals who underwent two PET measurements each with the same radioligand. The radioligands used were [^11^C]SCH23390 [27], [^11^C]AZ10419369 [28], [^11^C]PBR28 [29] and (R)-[^11^C]PK11195 [30]. Data from two target ROIs were selected as representative for each dataset. The two ROIs correspond to a region with higher and a region with lower specific binding for the radioligand used.

The [^11^C]SCH23390 cohort consisted of 15 male subjects [31]. [^11^C]SCH23390 binds to the dopamine D1 receptor, which is highly concentrated in the striatum, with a lower concentration in cortical regions and negligible expression in the cerebellum [32]. In this study, the target ROIs were the striatum and the frontal cortex.

The [^11^C]AZ10419369 cohort consisted of eight male subjects [33]. [^11^C]AZ10419369 binds to the serotonin 5-HT_1B_ receptor, which is highly concentrated in the occipital cortex, with a moderate concentration in the frontal cortex and negligible expression in the cerebellum. The occipital and frontal cortices were selected as the target ROIs for [^11^C]AZ10419369 [33].

The [^11^C]PBR28 cohort consisted of 6 males and 6 females [34] and the (R)-[^11^C]PK11195 cohort was comprised of 6 male individuals [35]. Both [^11^C]PBR28 and (R)-[^11^C]PK11195 bind to the 18 kDa translocator protein (TSPO), a proposed marker of glial cell activation [36–38]. TSPO has a widespread distribution across the whole brain, predominantly in grey matter [39]. In this study, the ROIs used for both TSPO ligands were the thalamus and the frontal cortex. Furthermore, arterial blood sampling, plasma measurements, and plasma metabolite analysis were performed and used in the analysis for the [^11^C]PBR28 and (R)- [^11^C]PK11195 cohorts as described previously [34, 35], as no true reference region is available for these radioligands.

### Kinetic modelling

A total of six commonly used kinetic models were used to quantify radioligand binding in the different datasets. For each analysis, both *kinfitr* (version 0.4.3) and PMOD (version 3.704, PMOD Technologies LLC., Zürich, Switzerland) were used. These estimates were subsequently compared to assess the correspondence between the two kinetic modelling tools. The same investigator (JT) performed the analysis with both tools.

For the quantification of [^11^C]SCH23390 and [^11^C]AZ10419369, the Simplified Reference Tissue Model (SRTM) [40], Ichise’s Multilinear Reference Tissue Model 2 (MRTM2) [41] and the non-invasive Logan plot [42] were used, with the cerebellum as a reference region for both radioligands. These models will be referred to as the “reference tissue models”, whose main outcome was binding potential (BP_ND_). Prior to performing MRTM2 and the non-invasive Logan plot, k_2_’ was estimated by fitting Ichise’s Multilinear Reference Tissue Model 1 (MRTM1) [41] for the TAC of the higher-binding region for each subject, the result of which was used as an input when fitting the models for all regions of that particular subject. The starting points, upper and lower bounds that were used for the nonlinear least squares models (2TCM and SRTM) are described in Supplementary Materials S1.

For the quantification of (R)-[^11^C]PK11195 and [^11^C]PBR28, the two-tissue compartment model (2TCM) [43–45], the Logan plot [46] and Ichise’s Multilinear Analysis 1 (MA1) [47] were used to estimate the volume of distribution (*V*_T_) using the metabolite-corrected arterial plasma (AIF) as an input function. These will henceforth be referred to as the “invasive models”. The delay between the TACs and arterial input function was fitted by the 2TCM using the TAC for the whole brain ROI. The default values in PMOD for the blood volume fraction (*v*_B_) were maintained throughout all analyses, which amounted to a *v*_B_ = 0 for MA1 and the invasive Logan plot and *v*_B_ = 0.05 for 2TCM. Default (constant) weighting was used in the analysis with PMOD, while the default weighting function options were used for *kinfitr* (described in Supplementary Materials S2).

The manner by which the analysis was performed was based on the explicit instructions provided along with each tool. However, when no explicit instructions were available, we inferred based on the instructions for previous analytical steps and the design of the user interface of each kinetic modelling tool to emulate best how users might actually use each tool. For instance, one difference between how both tools are used relates to the selection of t*, which is required when fitting the linearized models (MA1, MRTM2 and both invasive and non-invasive Logan plots). These linearized models rely on asymptotic approximations, and t* is the time point after which these approximations apply, and the curve can be described by the linear model. In *kinfitr*, a single t* value is selected by inspection of several plots as visual aids to maximise the number of frames (thereby limiting variance) without choosing too many, beyond the point of linearity (thereby resulting in bias) (detailed in Supplementary Materials S2) and used across individuals; while in PMOD, a unique t* value was selected for each individual PET measurement. In both cases, the design of the software makes it more difficult and time-consuming to do this the other way (more details provided in Supplementary Materials S2), and in the former case, this was a deliberate design decision to prevent over-fitting [21]. Importantly, the decision to focus on how the tools might be used in practice, rather than simply optimising the similarity of processing, provides more information about the extent to which outcomes might differ between tools, rather than the extent to which they might be made to be the same. We believe that this is of greater relevance to the research community. A separate analysis was performed for which the t* values fitted by PMOD and the weighting scheme used by PMOD were used in an analysis that was carried out using *kinfitr*, in order to investigate the effect which the differences in these parameters have on the differences between the tools. The t* values selected for the kinfitr analysis, and the median t* values fitted by PMOD, are provided in Supplementary Materials S3.

### Statistics

The primary aim of this study was to assess the correspondence between estimates of BP_ND_ (for reference tissue models) or *V*_T_ (for invasive models) obtained using *kinfitr* or PMOD, using a total of 6 different kinetic models in real data collected using four different radioligands. By using test-retest data, we were also able to evaluate the secondary aim of comparing the test-retest reliability within individuals for each tool. Test-retest data is subject to differences from one PET measurement to the next due to subtle biological changes or measurement error, so this is not a direct measure of accuracy. However, such a comparison allows for an indirect approximation of performance in cases where outcomes differ to a large extent.

The similarity between outcomes obtained using *kinfitr* and PMOD was evaluated using the Pearson correlation coefficient, the intraclass correlation coefficient (ICC), and bias.

The ICC represents the proportion of the total variance which is not attributable to measurement error or noise. Therefore, an ICC of 1 represents perfect agreement, while an ICC of 0 represents no signal and only noise. It is a measure of absolute agreement, i.e. even with a perfect correlation between outcomes, the ICC value will be penalised if there is a mean shift or if the gradient is not equal to 1. We used the ICC(A,1) [48], which is computed using the following equation:

$$ \mathrm{ICC}=\frac{\mathrm{M}{\mathrm{S}}_R-\mathrm{M}{\mathrm{S}}_E}{\mathrm{M}{\mathrm{S}}_R+\left(k-1\right)\mathrm{M}{\mathrm{S}}_E+\frac{k}{n}\left(\mathrm{M}{\mathrm{S}}_C-\mathrm{M}{\mathrm{S}}_E\right)} $$

where MS_R_ is the mean sum of squares of the rows, MS_*E*_ is the mean sum of squares of the error and MS_C_ is the mean sum of squares of the columns; and where *k* refers to the number of raters or observations per subject (in this case 2), and *n* refers to the number of subjects [49].

Bias was defined as the percentage change in the means of the values of the binding estimates. This measure was calculated as follows:

$$ \mathrm{Bias}=\frac{X_{kinfitr}-{X}_{\mathrm{PMOD}}}{X_{\mathrm{PMOD}}}\times 100\% $$

where *X* represents estimates of radioligand binding.

To compare the performance of each tool for assessing within- and between-subject variability, we calculated the mean, coefficient of variation (CV), ICC, within-subject coefficient of variation (WSCV) and absolute variability (AV).

The CV is calculated as a measure of dispersion. It is defined as follows:

$$ \mathrm{CV}=\frac{\hat{\sigma}}{\hat{\mu}}\times 100 $$%

Where $\hat{\sigma}$ represents the sample standard deviation and $\hat{\mu}$ the sample mean of the binding estimate value.

The ICC was calculated as above, since inter-rater agreement and test-retest reliability are both most appropriately estimated using the two-way mixed effects, absolute agreement, single rater/measurement ICC, the ICC(A,1) [50].

The within-subject coefficient of variation was calculated as a measure of repeatability and expresses the error as a percentage of the mean. It is calculated as follows:

$$ \mathrm{WSCV}=\frac{{\hat{\sigma}}_e}{\hat{\mu}}\times 100\% $$

where ${\hat{\sigma}}_e$ represents the standard error of the binding estimate value, which is analogous to the square root of the within subject mean sum of squares (MS_W_), which is also used in the calculation of the ICC above. $\hat{\mu}$ is the sample mean of the binding estimate value.

Finally, we also calculated the absolute variability (AV). This metric can be considered as an approximation of the WSCV above. While not as useful as the WSCV [51], AV has traditionally been applied within the PET field and is included for historical comparability.

$$ \mathrm{AV}=\frac{2\times \mid {X}_{\mathrm{PET}\ 1}-{X}_{\mathrm{PET}\ 2}\mid }{\mid {X}_{\mathrm{PET}\ 1}+{X}_{\mathrm{PET}\ 2}\mid}\times 100 $$

Where “*X*” refers to the value of the binding estimate and “PET 1” and “PET 2” refer to the first and second PET measurements in a test-retest experiment (in chronological order).

### Exclusions and deviations

All subjects in the [^11^C]SCH23390, [^11^C]AZ10419369 and (R)-[^11^C]PK11195 cohorts were included in the final analysis. However, one study participant belonging to the [^11^C]PBR28 cohort, was excluded due to exhibiting a poor fit in the PMOD analysis which resulted in an abnormally high *V*_T_ estimate (> 5 standard deviations from the mean of the rest of the sample, and a > 500% increase from the other measurement of the same individual) (Supplementary Materials S4). We were unable to resolve this problem using different starting, upper and lower limits.

Moreover, in the analysis of the [^11^C]PBR28 cohort, *kinfitr* returned warnings about high values of *k*_3_ and *k*_4_ for 2TCM in a total of 9 out of 48 TACs, of which 3 corresponded to the frontal cortex ROI and the remaining 6 were for the thalamus ROI. This is not entirely unexpected, as [^11^C]PBR28 is known to be slightly underfitted by this model [52], which increases the likelihood of local minima within the fitted parameter space. We also encountered this warning for the [^11^C]PK11195 cohort, with 8 warnings for 24 TACs, of which 2 corresponded to the frontal cortex ROI and the remaining 6 were from the thalamus. When parameter estimates are equal to upper or lower limit bounds, *kinfitr* returns a warning recommending either altering the bounds or attempting to use multiple starting points to increase the chance of finding the global minimum. Since in this case we deemed the values to be abnormally high, we opted for the latter strategy using the multiple starting point functionality of *kinfitr* using the *nls.multstart* package [53]. This entails setting a number of iterations to perform as an input function, and the software automatically fits each curve the given number of times (we selected 100) using randomly sampled starting parameters from across the parameter space, finally selecting the fit with the lowest sum of squared residuals. This process led to negligible changes in the *V*_T_ estimates, but yielded microparameter estimates whose values were no longer equal to the upper or lower limit bounds for [^11^C]PBR28. For the [^11^C]PK11195 cohort, the values remained at the parameter bounds; however, the parameter bounds were deemed to be reasonable given the distribution of the remainder of the data in lower ranges and were therefore left unchanged. We compared outcomes using both methods for both invasive radioligands for the two-tissue compartment model in Supplementary Materials S5, showing no differences for (R)-[^11^C]PK11195.

### Data and code availability

All analysis code is available at https://github.com/tjerkaskij/agreement_kinfitr_pmod. The data are pseudonymized according to national (Swedish) and EU legislation and cannot be fully anonymized, and therefore cannot be shared openly within this repository due to current institutional restrictions. Metadata can be openly published, and the underlying data can instead be made available upon request on a case by case basis as allowed by the legislation and ethical permits. Requests for access can be made to the Karolinska Institutet’s Research Data Office at rdo@ki.se.

## Results

**Fig. 1:**
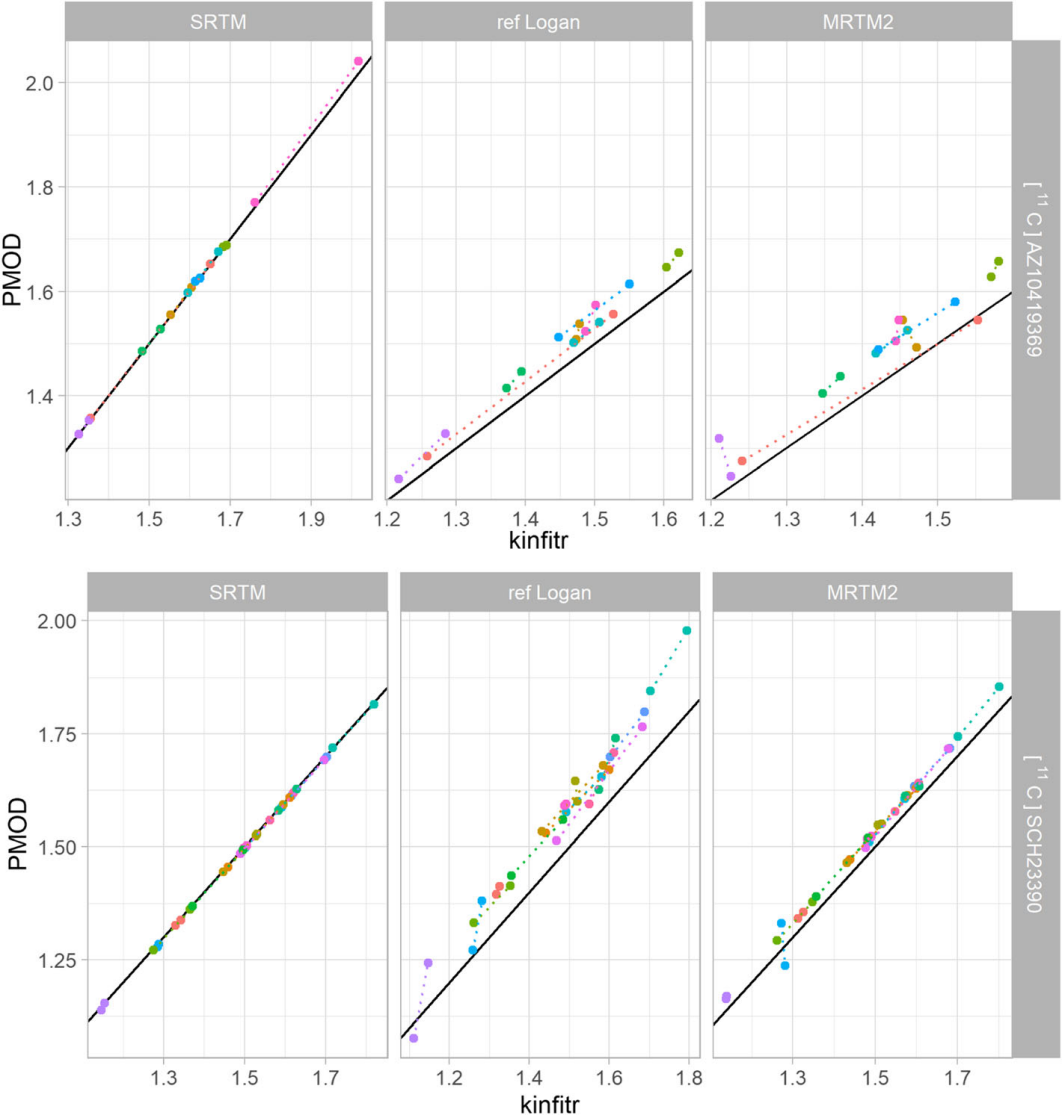
Comparison of BP_ND_ values calculated by kinfitr and PMOD. The relationship between binding estimates calculated by either *kinfitr* or PMOD. The results for the radioligand [^11^C]AZ10419369 are derived from the occipital cortex ROI, and for [^11^C]SCH23390, the striatum ROI. The diagonal line represents the line of identity. Each colour corresponds to a different subject, and the dotted lines connect both measurements from the same subject

**Fig. 2:**
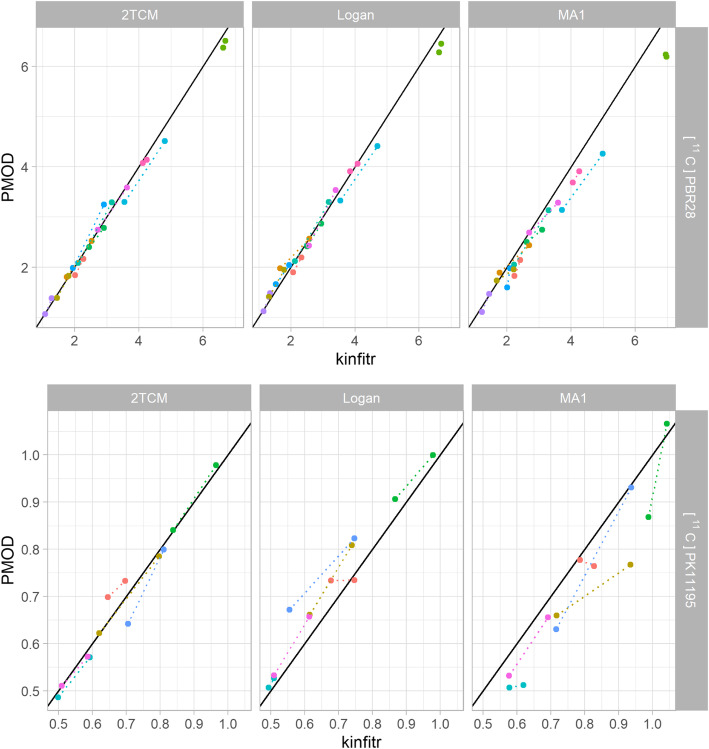
Comparison of *V*_T_ values calculated by kinfitr and PMOD. The relationship between binding estimates calculated by either *kinfitr* or PMOD. All results were derived from the frontal cortex region. The diagonal line represents the line of identity. Each colour corresponds to a different subject, and the dotted lines connect both measurements from the same subject

We found excellent correlations between *kinfitr* and PMOD, with a median Pearson's correlation coefficient of 0.99 (range 0.95–1.00) (Table 1). Likewise, we observed high absolute agreement between binding estimates computed using both tools, with a median ICC of 0.98 (range 0.80–1.00) (Table 1, Figs. 1 and 2, Supplementary Materials S6) [51]. It was observed that the linearized methods (i.e. MA1, MRTM2 and both invasive and non-invasive Logan plots) generally exhibited lower agreement than the non-linear models. We also ran the linearized models in kinfitr using the t* values fitted by PMOD, which resulted in slight improvements in correlation (mean pairwise increase of 0.001), ICCs (mean pairwise increase of 0.004) and decreased bias (mean pairwise decrease of 0.7%) (Supplementary Materials S7).

**Table 1:** Correspondence between *kinfitr* and PMOD

		Pearson’s *r*		ICC		Bias (%)	
Ligand	Model	Region 1	Region 2	Region 1	Region 2	Bias 1	Bias 2
Invasive							
[^11^C]PBR28	2TCM	1.00	1.00	0.99	1.00	2.01	1.19
	Logan	1.00	1.00	0.99	0.99	0.47	− 0.27
	MA1	1.00	0.99	0.95	0.97	10.07	9.55
[^11^C]PK11195	2TCM	1.00	0.98	1.00	0.98	1.16	0.69
	Logan	1.00	0.97	0.99	0.94	− 3.40	− 5.88
	MA1	0.99	0.95	0.97	0.89	4.99	9.54
Non-invasive							
[^11^C]AZ10419369	SRTM	1.00	1.00	1.00	1.00	-0.17	0.13
	ref Logan	0.99	0.99	0.93	0.91	− 2.95	− 3.55
	MRTM2	0.97	0.96	0.86	0.80	− 3.94	− 5.35
[^11^C]SCH23390	SRTM	1.00	1.00	1.00	1.00	0.24	0.53
	ref Logan	0.99	0.99	0.88	0.96	− 5.26	− 4.31
	MRTM2	1.00	1.00	0.98	0.99	− 2.07	− 2.17

Region 1 corresponds to the occipital cortex for the radioligand [^11^C]AZ10419369, the striatum for [^11^C]SCH23390 and the thalamus for both (R)-[^11^C]PK11195 and [^11^C]PBR28. Region 2 corresponds to the frontal cortex for all four radioligands which were used in this study. Abbreviations: *2TCM* two-tissue compartmental model, *Logan* invasive Logan plot, *MA1* Ichise’s Multilinear Analysis 1, *SRTM* simplified reference tissue model, *ref Logan* reference tissue Logan plot, *MRTM2* Ichise's Multilinear Reference Tissue Model 2 (MRTM2), *ICC* intra-class correlation coefficient, *Pearson’s r* Pearson’s correlation coefficient

We also found strong correlations between the binding estimates of the different kinetic models that were estimated using *kinfitr* and PMOD (Supplementary Materials S8). When comparing the binding estimates of the three reference tissue models within *kinfitr* and PMOD, respectively, there was a median Pearson’s correlation coefficient of 0.99 for both tools (range 0.76–1.00 for PMOD and 0.71–1.00 for *kinfitr*). For the invasive models, there was a median Pearson’s correlation coefficient of 0.98 for PMOD (range 0.53–1) and 0.98 for *kinfitr* (range 0.92–1). When using the t* values fitted by PMOD in the *kinfitr* analysis, we observed a median Pearson’s correlation coefficient of 0.99 (range 0.68–1) between the non-invasive models and a median Pearson’s correlation coefficient of 1.0 (range 0.93–1) for the invasive models.

Both tools performed similarly in terms of test-retest reliability, with no substantial differences seen in the mean values, dispersion (CV), reliability (ICC) or variability (WSCV and AV) (Supplementary Materials S9).

### Microparameters

**Fig. 3:**
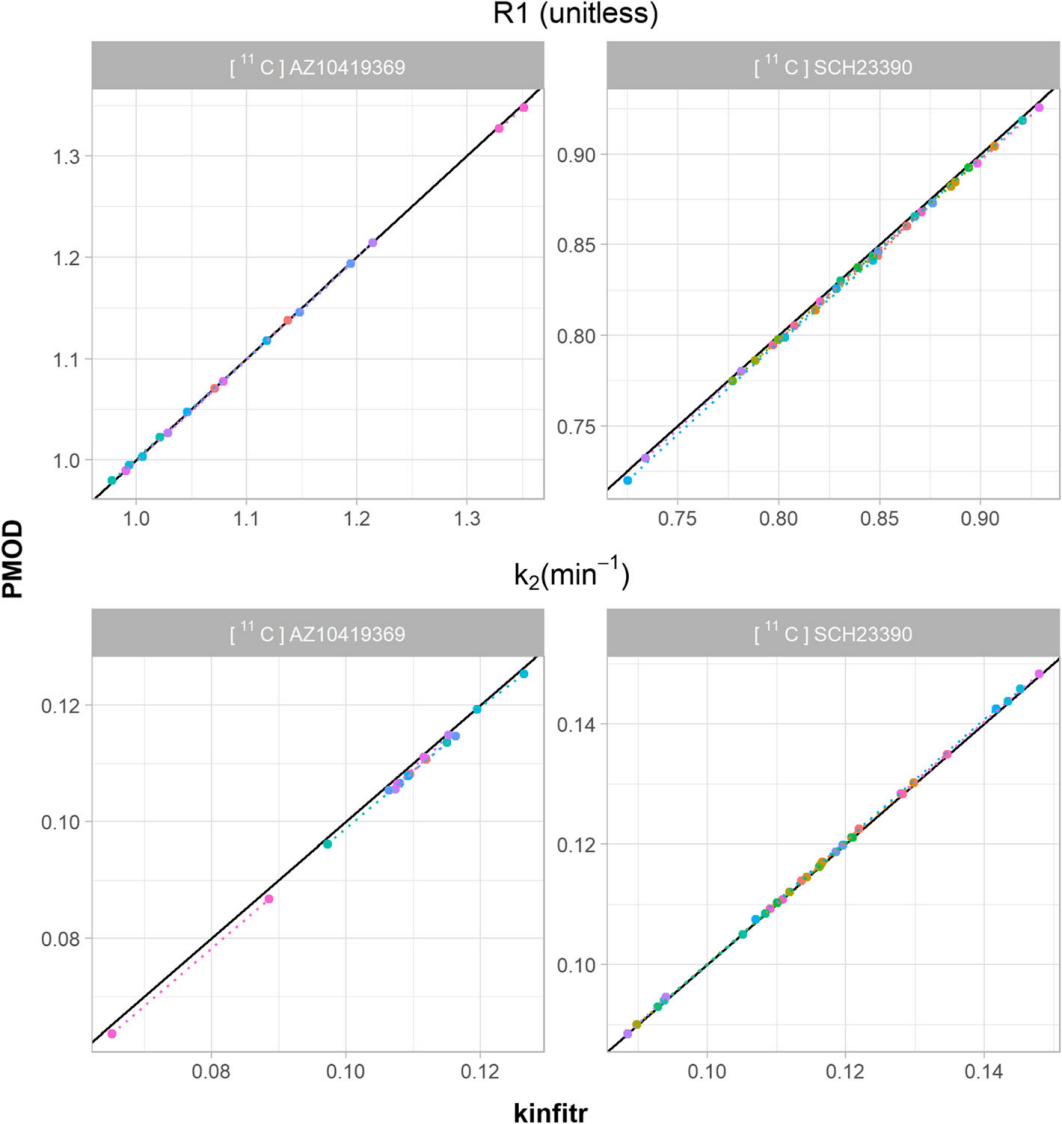
Microparameter comparison for the simplified reference tissue model (SRTM). The relationship between the values of individual rate constants calculated by either kinfitr or PMOD. The results for the radioligand [^11^C]AZ10419369 are derived from the occipital cortex ROI, whereas the results for [^11^C]SCH23390 correspond to the striatum. The diagonal line represents the line of identity. Each colour corresponds to a different subject, and the dotted lines connect both measurements from the same subject

**Fig. 4:**
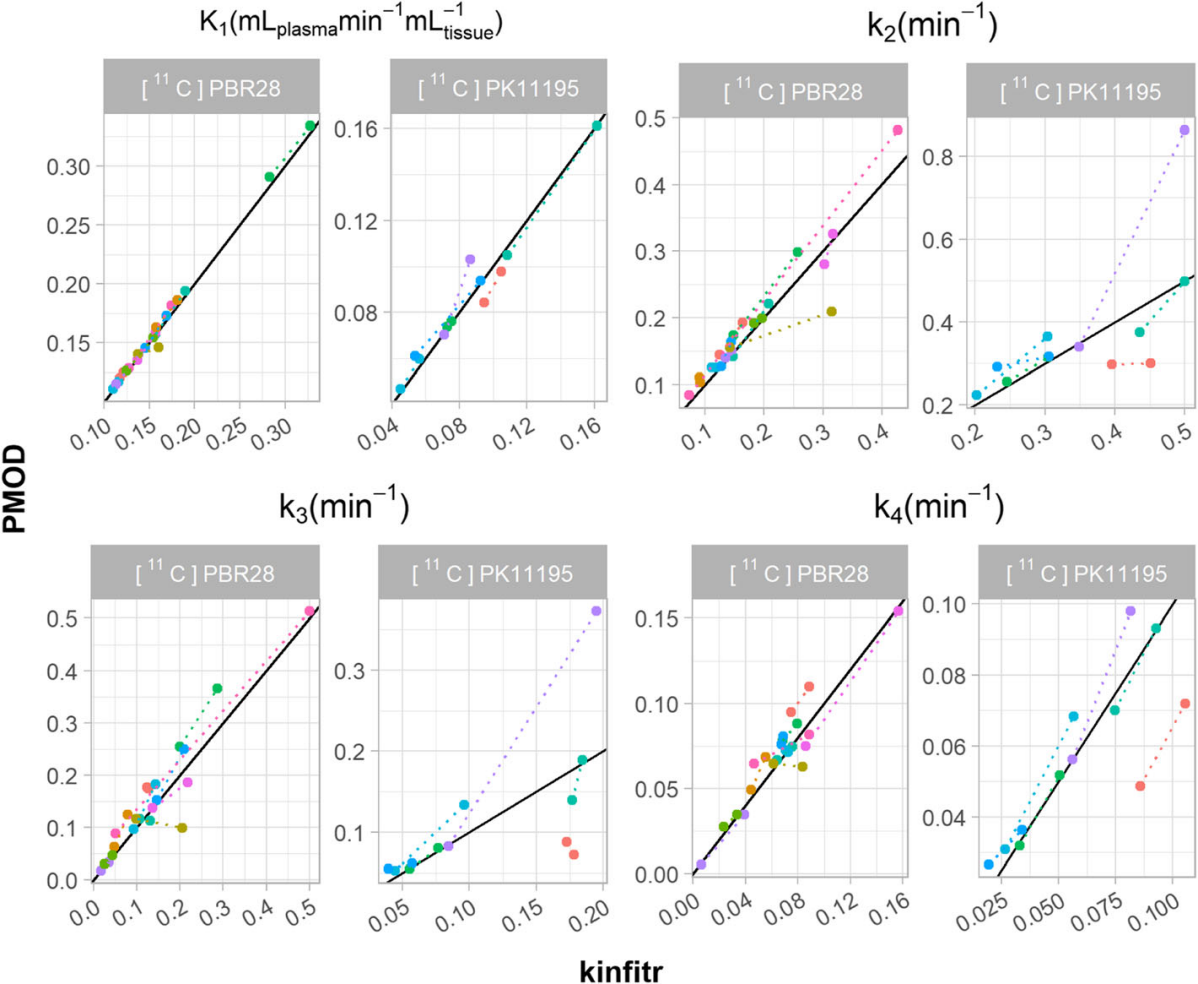
Microparameter comparison for the two-tissue compartment model (2TCM). The relationship between the values of individual rate constants calculated by either kinfitr or PMOD. All results were derived from the thalamus region. The diagonal line represents the line of identity. Each colour corresponds to a different subject, and the dotted lines connect both measurements from the same subject

We also compared the values of microparameters (i.e. individual rate constants) estimated using the non-linear methods. Figure 3 shows a comparison between the values of *R*1 and *k*_2_ obtained using SRTM for [^11^C]AZ10419369 and [^11^C]SCH23390. We observed Pearson’s correlation coefficients of > 0.99 for both *R*1 and *k*_2_ estimated by *kinfitr* and PMOD. Similarly, the relationships between the microparameter estimates obtained using 2TCM for [^11^C]PBR28 and (R)-[^11^C]PK11195 were assessed (Fig. 4). We found high correlations between *kinfitr* and PMOD estimates of *K*_1_, *k*_2_, *k*_3_ and *k*_4_ (mean Pearson’s correlation coefficients of 0.99, 0.81, 0.80, and 0.88, respectively).

## Discussion

In this study, we evaluated the performance of *kinfitr* by comparing radioligand binding estimates to those obtained with the established commercial software PMOD. We assessed the similarity between these tools using four datasets, each encompassing a different radioligand, and employed three kinetic models for invasive and non-invasive applications. Mean regional BP_ND_ and *V*_T_ values computed by both tools were similar to those reported in previous literature on the same radioligands [33–35, 54]. We observed high correspondence between estimates of BP_ND_ and *V*_T_ using *kinfitr* and PMOD. Furthermore, there were no substantial differences between the tools in terms of test-retest reliability for these measures. We further found that both tools exhibited a high correspondence between estimates of the microparameters, as well as between the macroparameter estimates of the different models assessed using each tool separately. While the bias between some outcome measures estimated with the two tools was non-negligible (Table 1), the high correlations for all outcomes mean that this would not present an issue when using one or the other tool within a given dataset.

Despite the overall high similarity with regard to binding estimates, the linearized models (i.e. MA1, MRTM2 and both invasive and non-invasive Logan plots) exhibited a slightly lower degree of agreement the nonlinear models (2TCM and SRTM). This observation is partially explained by the fact that the linearized models require the selection of a t* value, which was performed differently using the two tools, and the correspondence between the tools improved slightly overall when using the t* values fitted by PMOD in the analysis in *kinfitr*. As described in more detail in the Supplementary Material S2, PMOD fits a t* value for the user, whereas *kinfitr* requires the user to specify a t* based on several plots as visual aids with which to select an appropriate value. As such, the PMOD interface makes it more convenient to fit t* values independently for each individual, while the *kinfitr* interface encourages selecting a single t* value which is applicable across all study participants.

With regard to the user interface of the two tools, the most important difference is that *kinfitr* requires the user to interact with the data using code, while PMOD makes use of a graphical user interface (GUI), i.e. the user clicks buttons and selects items from drop-down menus. As such, *kinfitr* requires learning basic R programming before it can be used effectively, while PMOD can essentially be used immediately. Therefore, *kinfitr* may be perceived as having a steeper learning curve than PMOD. However, in our experience, *kinfitr* provides the user with greater efficiency once a moderate degree of proficiency has been gained. For instance, as a result of the code interface, re-running an analysis using *kinfitr* on all study participants using different parameters (e.g. altering a fixed *v*_B_ or t* value) or a different model, can be performed by modifying only the relevant lines of code. In contrast, performing re-analyses using PMOD can require a great deal of manual effort, as all tasks must essentially be repeated. This exemplifies the fundamental benefit of computational reproducibility: by crystallising all steps in computer code, the results can easily be generated anew from the raw input data. This procedure also makes the detection of potential errors substantially easier as all user actions are recorded transparently in the analysis code and allows others to more quickly and easily adapt, modify or build upon previous work.

Another important consideration when comparing different tools is the time and effort required to transition from one tool to another due to file formats or structure. For the PMOD analysis, TAC data was formatted according to the PMOD structure, while *kinfitr* does not make any requirements about the format of the input data other than that the TACs are expressed in numeric vectors. Importantly, the recent development of the Brain Imaging Data Structure (BIDS) [55] and its PET extension (BEP009) has now been established as the standard for how PET image and blood data should be organised and shared in the recent consensus guidelines [13]. This is expected to simplify the use of and design of new tools for analysis of PET data greatly. Both k*infitr* and PMOD, according to its documentation, support the BIDS standard for ancillary data (i.e. not originating from the PET image itself, such as blood data and injected radioactivity). In this study, TACs were used which are not currently part of the BIDS structure as they are derived from PET images following image processing; however, another BIDS standard for PET Pre-processing derivatives (BEP023) is currently under development.

It is important to note that the kinetic modelling was not performed in an identical manner between the two tools; rather we performed the modelling in a manner as consistent with the way users might actually use the software as possible. This was done in order to emphasize ecological validity. While this diminishes the extent to which we can specifically compare the outcomes using both of the two tools, our intention was instead to compare how both tools would be expected to perform independently in practice. This approach focuses on the extent to which outcomes might potentially differ between these tools, rather than the extent to which they can be made similar. It is reasonable to assume that even higher agreement could be achieved if additional measures were taken to make each analytic step identical. We observed slightly increased correspondence when running the *kinfitr* analyses using the PMOD t* values (although paradoxically not PMOD weights) (Supplementary Materials S7), but additional measures such as using identical delay, *k*2’ values, integration algorithms, interpolation and starting values could all impact the correspondence between tools.

As we assessed the correspondence between these tools using real data, we were unable to directly compare their accuracy. Our aim in this study was instead to ascertain that both tools perform similarly in an applied setting using real data, given all its imperfections—“warts and all”. Furthermore, by including test-retest data, we were able to examine the question of accuracy indirectly—although this data is subject to both biological and measurement-related differences between PET measurements. One method by which to evaluate accuracy directly would be to compare performance using simulated data. However, given the high degree of correspondence between the tools, any differences observed using simulated data would be strongly dependent on correspondence of the data-generating process with the model being applied and its assumptions. Hence, if we simulate data using one tool and its particularities, then this tool will have an unfair advantage in modelling the simulated data, limiting the relevance of such a comparison. A future study making use of carefully simulated data using different tools or methods would be of some relevance for the field to compare the accuracy and performance of PET analysis tools.

## Conclusions

In summary, we showed good correspondence between the open-source R package *kinfitr*, and the widely used commercial application PMOD, which we have treated as the reference point. We, therefore, conclude that *kinfitr* is a valid and reliable tool for kinetic modelling of PET data.

## Supplementary information

**Additional file 1: Supplementary Materials S1** Parameter fitting details. **Supplementary Materials S2** Differences Between the analyses done in PMOD and *kinfitr.***Supplementary Materials S3** t* values. **Supplementary Materials S4** Demonstrating the outlier detected in the PBR28 analysis relative to the remainder of the data. **Supplementary Materials S5** The effect of iteration over starting points on the binding estimates using 2TCM with *kinfitr.***Supplementary Materials S6** Binding estimates. **Supplementary Materials S7** Agreement between kinfitr and PMOD for linearised models when using the t* values fitted by PMOD and constant weights in the analysis run using kinfitr. **Supplementary Materials S8** Relationship between Binding Outcomes between Models Estimated Using Each Tool. **Supplementary Materials S9** Test-retest analysis.

## Data Availability

All analysis code is available at https://github.com/tjerkaskij/agreement_kinfitr_pmod. The data are pseudonymized according to national (Swedish) and EU legislation and cannot be fully anonymized, and therefore cannot be shared openly within this repository due to current institutional restrictions. Metadata can be openly published, and the underlying data can instead be made available upon request on a case by case basis as allowed by the legislation and ethical permits. Requests for access can be made to the Karolinska Institutet’s Research Data Office at rdo@ki.se.

## Abbreviations

PET: Positron emission tomography
ROI: Region of interest
TAC: Time-activity curve
TSPO: 18 kDa translocator protein
SRTM: Simplified Reference Tissue Model
MRTM2: Ichise’s Multilinear Reference Tissue Model 2
BP_ND_: Binding potential
MRTM1: Ichise’s Multilinear Reference Tissue Model 1
2TCM: Two-tissue compartment model
MA1: Ichise’s Multilinear Analysis 1
*V*_T_: Volume of distribution
AIF: Arterial input function
*v*_B_: Blood volume fraction
ICC: Intraclass correlation coefficient
CV: Coefficient of variation
WSCV: Within-subject coefficient of variation
AV: Absolute variability
