# Supplementary material for: Kinfitr — an open-source tool for reproducible PET modelling: validation and evaluation of test-retest reliability
Source: EJNMMI Res. 2020 Jul 8;10:77. doi: 10.1186/s13550-020-00664-8 (PMC7343683; doi:10.1186/s13550-020-00664-8)
Supplement: Supplementary file 1 — Additional file 1: Supplementary Materials S1 Parameter fitting details. Supplementary Materials S2 Differences Between the analyses done in PMOD and kinfitr.Supplementary Materials S3 t* values. Supplementary Materials S4 Demonstrating the outlier detected in the PBR28 analysis relative to the remainder of the data. Supplementary Materials S5 The effect of iteration over starting points on the binding estimates using 2TCM with kinfitr.Supplementary Materials S6 Binding estimates. Supplementary Materials S7 Agreement between kinfitr and PMOD for linearised models when using the t* values fitted by PMOD and constant weights in the analysis run using kinfitr. Supplementary Materials S8 Relationship between Binding Outcomes between Models Estimated Using Each Tool. Supplementary Materials S9 Test-retest analysis. [file 13550_2020_664_MOESM1_ESM.pdf]

Supplementary material

Supplementary Materials S1

Parameter fitting details

Both kinfitr and PMOD make use of the Levenberg-Marquard algorithm for nonlinear least-squares estimation (in SRTM and 2TCM). This algorithm requires starting values and upper and lower bounds for all fitted parameters.

Two Tissue Compartment Model: In kinfitr, the starting values (and their upper and lower limits) are as follows: K1, k2, k3, k4=0.1 (0.0001 – 0.5), vB=0.05 (0.01 – 0.1), delay=0 (-0.5 – 0.5). In PMOD, these are K1and k2 = 0.1 (0 – 8); k3 and k4 = 0.01 (0 – 8), vB = 0.05 (0-1.0), delay = 0 (-2 - 2).

Simplified Reference Tissue Model: In kinfitr, the starting values (and their upper and lower limits) are as follows: R1=1 (0 – 10), k2=0.1 (0 – 1), BP_{ND}=1.5 (-10 – 15). In PMOD, these are R1=0.78 (0 – 10), k2=0.1 (0 – 1), BP_{ND}= 1 (0 – 20).

t* values: The t* values are provided in supplementary materials S3. In PMOD, these were automatically assigned. In kinfitr, these were selected using the t*-finder figures provided in supplementary materials S2.

Supplementary Materials S2

Differences Between the analyses done in PMOD and *kinfitr*

PMOD analysis

Interface and Auditability

PMOD relies on a graphical user interface (GUI) (Figure 1). Data is sequentially loaded for each study participant, one-by-one, and the kinetic models are fitted using buttons and drop-down menus. User actions can be logged by purchasing the "audit trail license" (1).

Weighting schemes

We made use of the default weighting scheme of PMOD, which is constant weighting. This means that the same weight is applied to all data points. The whole brain TAC was used for the calculation of weights.

t* values

For the selection of a value for t*, PMOD allows the user to automatically select an appropriate value. By default, the software will search for the earliest value of t* such that the deviation between the regression line and all of the observed values is less than 10% (2). The t* value was fitted using the higher-binding region for each tracer. This t* value was then used for the other regions for each tracer and model.

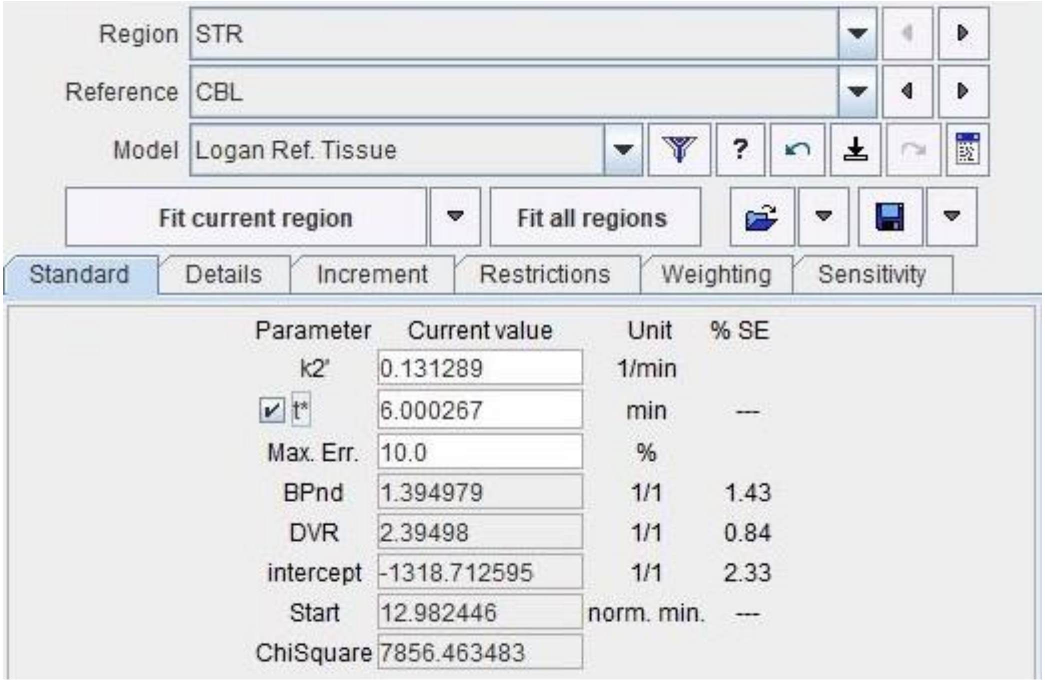

This image shows the PMOD interface during the fitting of the reference tissue Logan plot for a study participant belonging to the [^{11}C]SCH23390 cohort. The t button is checked, indicating that t* will be or has just been fitted. The Max.Err criterion, set to 10%, indicates the cutoff value of maximum percentage residuals used to fit t*. The Striatum, being a high-binding region for [^{11}C]SCH23390, was used when fitting t* for all study participants belonging to that cohort, and the cerebellum was used as a reference region. k2' was manually inserted after having estimated it using MRTM1.*

PMOD is therefore designed in such a way that fitting a t* value for each PET examination is made very convenient. If one were to wish to use a single t* value across multiple individuals based on the results from everyone, this would take more time and effort. One would sequentially load in the data for each subject individually, fit the model and t* value, and write down the different t* values obtained. Afterward, one could evaluate these values and decide on an appropriate value for t*, and then sequentially load in the data for each subject once more, manually enter the t* value, and fit the model again using this value. For this reason, we suggest that the user interface encourages the use of individual t* values for each measurement, as this approach is much more convenient.

kinfitr

Interface and Auditability

In *kinfitr*, the user interacts with the software through R code. We made use of R notebooks, by which writing, code and code outputs are all interspersed with one another (3). These analysis notebooks are shared online (https://github.com/tjerkaskij/agreement_kinfitr_pmod). All user actions are therefore represented in the analysis code and can be checked and audited directly. This is one of the central benefits of computational reproducibility.

One of the other proposed benefits of computational reproducibility is that code can be recycled and repurposed from project to project, or even between analysts. An example of this in practice is that the code within the analysis notebooks for [^{11}C]AZ10419369 and [^{11}C]SCH23390 is nearly identical and could, therefore, be copied from one to the other analysis and modified as necessary.

Weighting schemes

We used the default weighting scheme from *kinfitr*. This method defines weights as the square root of the product of the frame durations and the non-decay-corrected time-activity curve of a large region. The resulting values are then taken as a proportion of the range between 0.7 and 1 to restrict their range. The whole brain TAC was used for the calculation of weights. Weights were applied for all models in kinfitr apart from the Logan and non-invasive Logan models, owing to their making use of transformed values for the predicted value. For these cases, uniform weights were used.

t* Values

In contrast to PMOD, the selection of t* is not automated in *kinfitr*. Rather, the user is presented with R^2 values, maximum percentage residuals and changes in binding estimates for each potential value of t*, for three different regions of interest for each PET measurement, recommended to consist of regions with high, medium and low binding (see figure below). In this way, the user must select an appropriate value for t* by examining all of the available information. It is further explicitly recommended in the documentation that

these visual aids should be examined for several individuals/measurements to select a single t* value for the entire cohort.

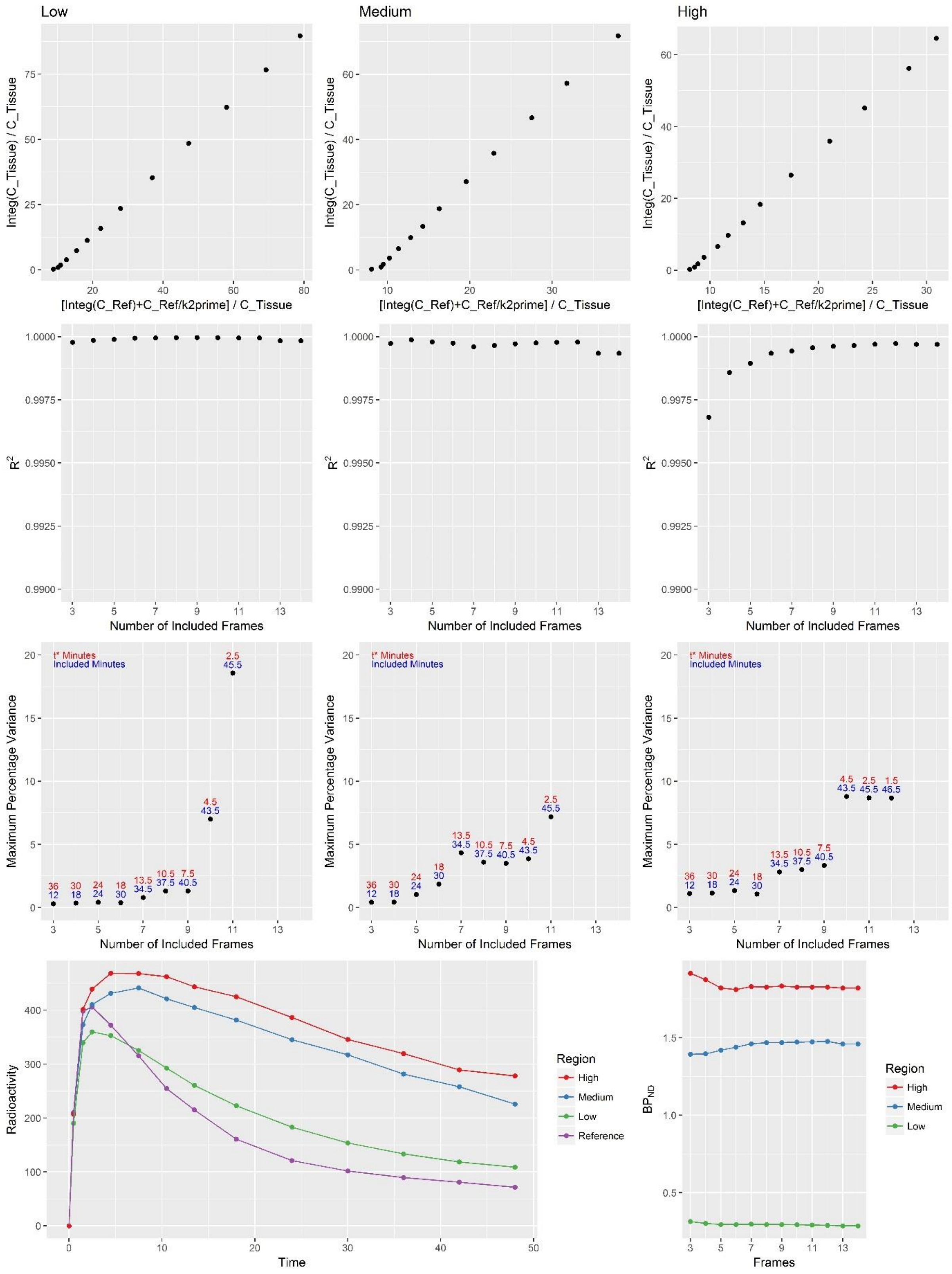

Graphical aids provided by kinfitr for the selection of an appropriate t value.*

In *kinfitr*, all data is loaded at once, and so producing all t* selection figures for several or all measurements in a cohort is straightforward. As such, *kinfitr* makes it more convenient to

examine these t* visual aids and to make a judgement about the most appropriate value for a t*, across the whole cohort. If, instead, one were to wish to select an individual value for t* for each individual, this would take a great deal more time and effort. One would have to examine the t* aids one-by-one, and write down the selected t* value for each, add the selected t* values to the data, and then re-run the modelling by pointing the model to the newly created t* column for which value to use. Instead, this was a deliberate and opinionated design decision to avoid the potential for overfitting the selection of t* values, and to present the user with as much information as possible to make as informed a choice as possible. For this reason, the user interface encourages the use of a single t* values for a whole cohort, as this approach is much more convenient, and is furthermore explicitly encouraged.

Supplementary Materials S3

t* values fitted by PMOD for the invasive models

		Logan				MA1			
**Ligand**	**Region**	**Median**	**IQR**	**Min**	**Max**	**Median**	**IQR**	**Min**	**Max**
[^{11}C]PBR28	FC	15.14	6.98	7.18	33.33	7.49	10.47	3.75	27.5
[^{11}C]PK11195	FC	15.00	3.00	9.00	21.00	15.00	4.50	9.00	27.0
[^{11}C]PBR28	THA	15.14	6.98	7.18	33.33	7.49	10.47	3.75	27.5
[^{11}C]PK11195	THA	15.00	3.00	9.00	21.00	15.00	4.50	9.00	27.0

The t value is displayed in terms of the number of frames counted from the final frame. Abbreviations: FC = Frontal cortex, THA = Thalamus, MA1 = Ichise's Multilinear Analysis 1. IQR = inter-quartile range*

t* values fitted by PMOD for the reference tissue models

		ref Logan				MRTM2			
**Ligand**	**Region**	**Median**	**IQR**	**Min**	**Max**	**Median**	**IQR**	**Min**	**Max**
[^{11}C] AZ10419369	FC	5.29	2.03	2.6	9.23	1.18	0.31	0.9	1.67
[^{11}C] SCH23390	FC	6.00	0.00	2.0	15.00	0.00	0.00	0.0	2.00
[^{11}C] AZ10419369	OC	5.29	2.03	2.6	9.23	1.18	0.31	0.9	1.67
[^{11}C] SCH23390	STR	6.00	0.00	2.0	15.00	0.00	0.00	0.0	2.00

The t value is displayed in terms of the number of frames counted from the final frame. Abbreviations: FC = Frontal cortex, OC = Occipital cortex, STR = Striatum, ref Logan = reference tissue Logan kinetic model, MRTM2 = Ichise's Multilinear Reference Tissue Model 2, IQR = inter-quartile range.*

t* values selected for the kinfitr analysis.

Radioligand	Model	t*
[^{11}C]PBR28	MA1	21.483
	Logan	9.483
[^{11}C]PK11195	MA1	19.000
	Logan	9.000
[^{11}C]SCH23390	MRTM2	0.000
	Reference tissue Logan	2.000
[^{11}C]AZ10419369	MRTM2	2.683
	Reference tissue Logan	9.350

The t* values are displayed in minutes. Abbreviations: MA1 = Ichise's Multilinear Analysis 1, ref Logan = reference tissue Logan kinetic model, MRTM2 = Ichise's Multilinear Reference Tissue Model.

Supplementary Materials S4

Demonstrating the outlier for PBR28 VT relative to the remainder of the data

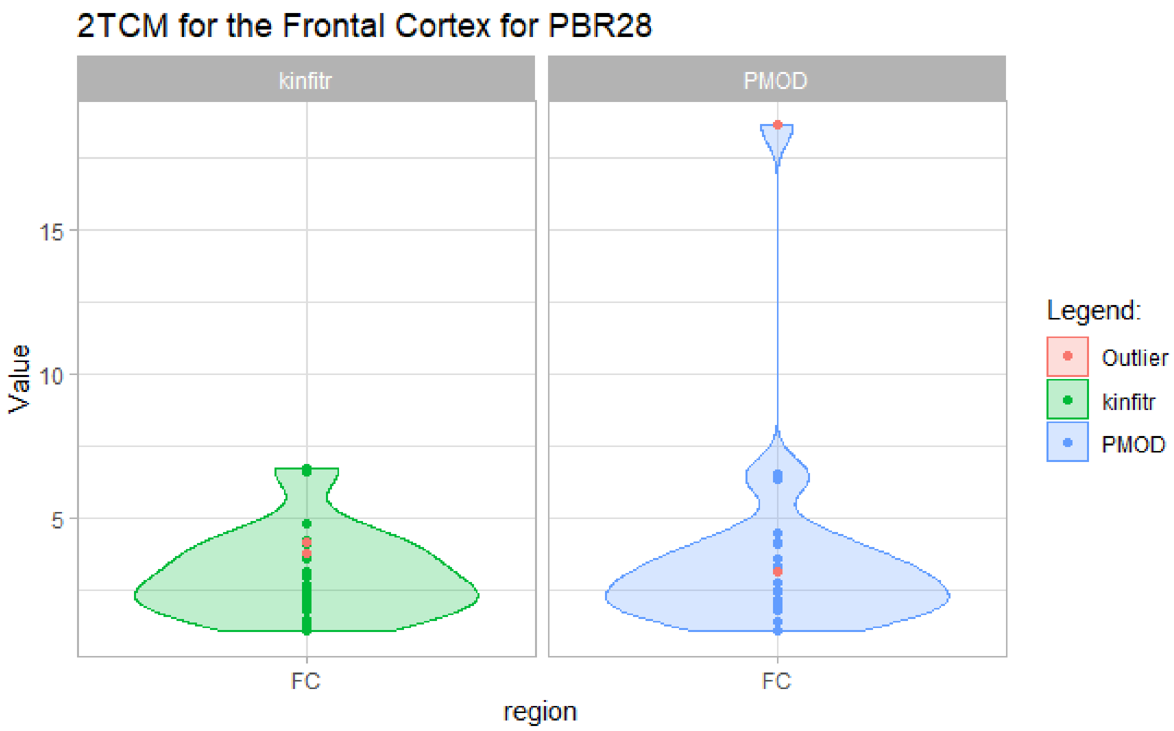

Violin plot showing the results for 2TCM for the frontal cortex using the radioligand PBR28. The excluded study participant is marked in red. Results are shown for both kinetic modelling t

Supplementary Materials S5

The effect of iteration over starting points on the binding estimates using 2TCM with *kinfitr*

Agreement between 2TCM using kinfitr with and without iteration

		Pearson's r		ICC		Bias (%)	
**Ligand**	**Model**	**Thalamus**	**Frontal cortex**	**Thalamus**	**Frontal cortex**	**Thalamus**	**Frontal cortex**
[^{11}C]PBR28	2TCM	0.99	0.99	0.99	0.99	2.13	5.4
[^{11}C]PK11195	2TCM	1.00	1.00	1.00	1.00	0.00	0.0

Abbreviations: ICC = intra-class correlation coefficient.

V_T values computed using 2TCM with kinfitr for the radioligands [^{11}C]PBR28 and [^{11}C]PK11195 with and without iteration.

Ligand	Region	Iteration	Median	IQR	Min	Max
[^{11}C]PBR28	FC	Yes	2.813	1.850	1.083	6.697
		No	2.813	1.681	0.917	6.697
	THA	Yes	3.234	2.268	1.334	9.444
		No	3.234	2.207	1.334	9.444
[^{11}C]PK11195	FC	Yes	0.672	0.208	0.500	0.966
		No	0.672	0.208	0.500	0.966
	THA	Yes	0.712	0.226	0.505	1.203
		No	0.712	0.226	0.505	1.203

Supplementary Materials S6

Binding estimates.

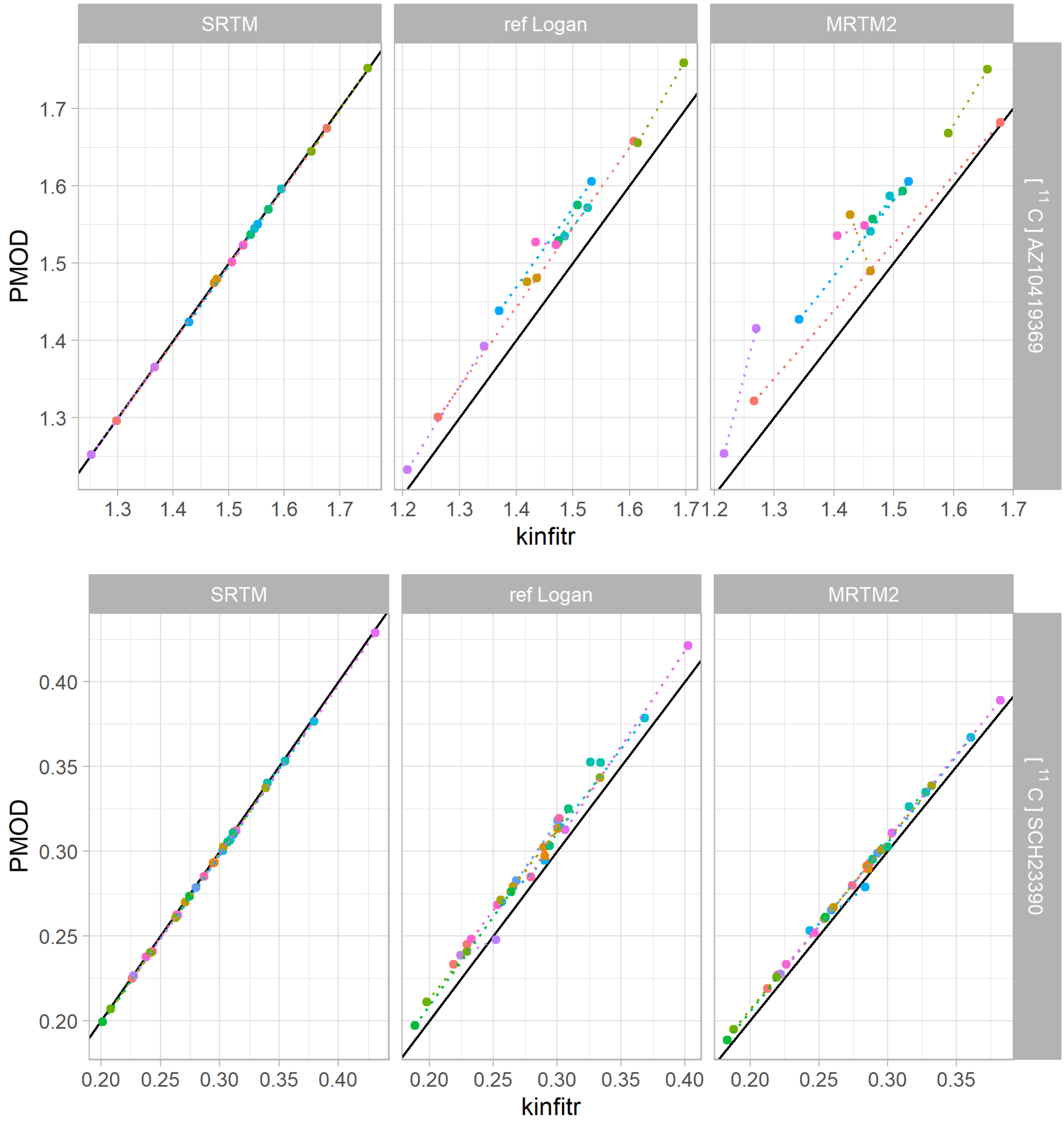

Comparison of BP_{ND} values calculated by kinfitr and PMOD. The relationship between binding estimates calculated by either *kinfitr* or PMOD All results were derived from the frontal cortex region. The diagonal line represents the line of identity. Each colour corresponds to a different subject, and

the dotted lines connect both measurements from the same subject.

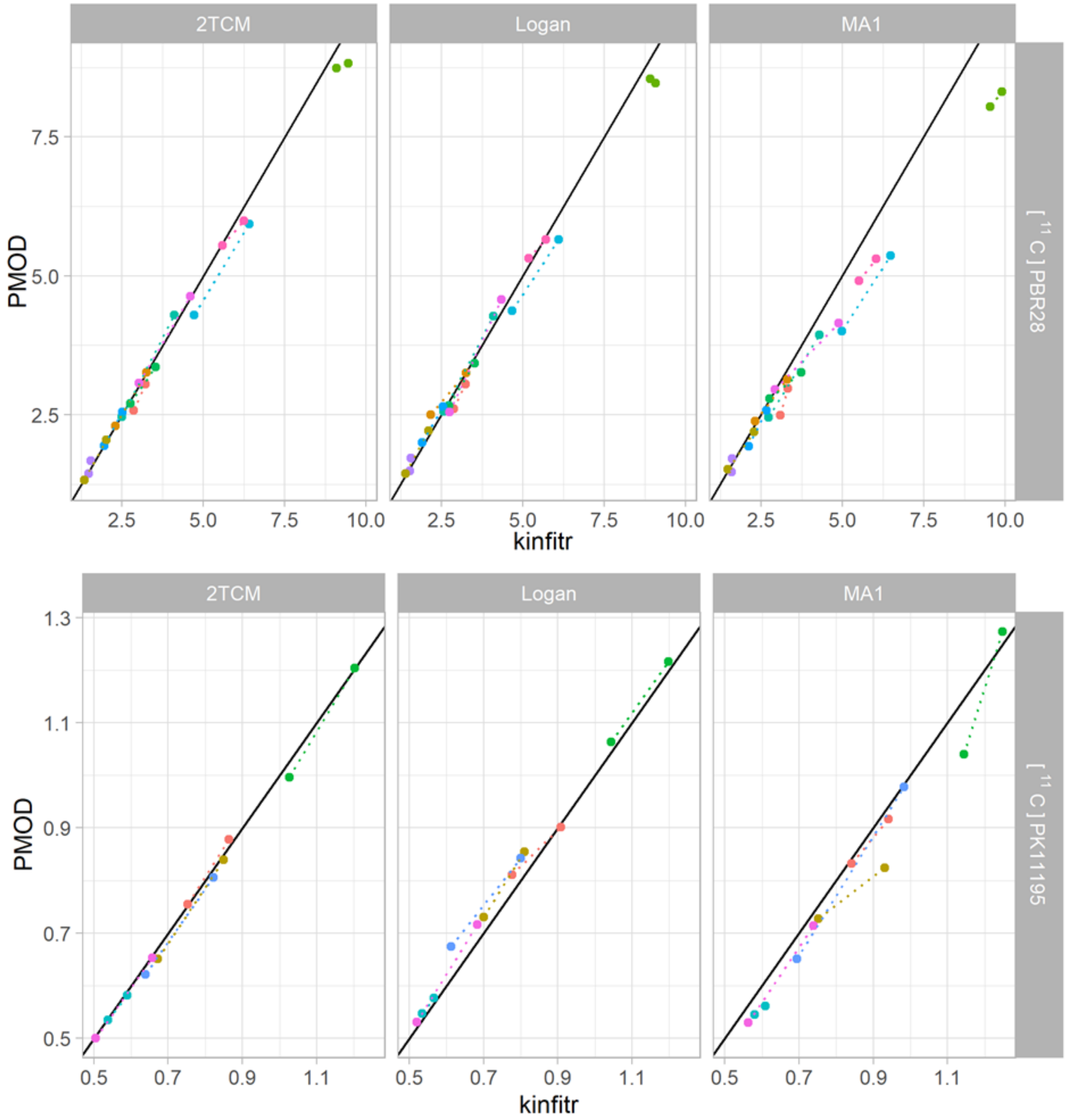

Comparison of V_T values calculated by kinfitr and PMOD. The relationship between binding estimates calculated by either *kinfitr* or PMOD All results were derived from the thalamus region. The diagonal line represents the line of identity. Each colour corresponds to a different subject, and the dotted lines connect both measurements from the same subject.

Supplementary Materials S7

Agreement between kinfitr and PMOD for linearised models when using the PMOD t* values and weights in kinfitr

Agreement between kinfitr and PMOD using fitted or selected t* values for the invasive models

			Pearson's r		ICC		Bias (%)	
**Ligand**	**Model**	**t***	**Region 1**	**Region 2**	**Region 1**	**Region 2**	**Bias 1**	**Bias 2**
[11C]PBR28	Logan	Selected	1.00	1.00	0.99	0.99	1.06	0.37
		Fitted	1.00	0.99	0.97	0.97	7.91	9.92
	MA1	Selected	1.00	1.00	0.95	0.97	10.53	10.06
		Fitted	1.00	0.99	0.98	0.97	7.84	9.30
[11C]PK11195	Logan	Selected	1.00	0.97	0.99	0.94	-3.26	-5.68
		Fitted	1.00	1.00	1.00	1.00	0.07	0.02
	MA1	Selected	0.99	0.95	0.97	0.89	5.07	9.63
		Fitted	1.00	1.00	1.00	1.00	-0.20	-0.30

Region 1 corresponds to the thalamus for both (R)- [11C]PK11195 and [11C]PBR28. Region 2 corresponds to the frontal cortex. Abbreviations: “2TCM” = Two-tissue compartmental model, “Logan” = Invasive Logan plot, “MA1” = Ichise’s Multilinear Analysis 1, “ICC” = intra-class correlation coefficient, “Pearson’s r” = Pearson’s correlation coefficient.

Agreement between kinfitr and PMOD using fitted or selected t* values for the non-invasive models

			**Pearson's r**		**ICC**		**Bias (%)**	
**Ligand**	**Model**	**t***	**Region 1**	**Region 2**	**Region 1**	**Region 2**	**Bias 1**	**Bias 2**
$[^{11}C]$AZ10419369	ref Logan	Selected	0.99	0.99	0.93	0.91	-2.93	-3.53
		Fitted	0.98	0.98	0.88	0.89	-3.84	-3.87
	MRTM2	Selected	0.97	0.96	0.87	0.81	-3.84	-5.24
		Fitted	0.95	0.95	0.81	0.80	-4.76	-5.28
$[^{11}C]$SCH23390	ref Logan	Selected	0.99	0.99	0.90	0.97	-4.81	-4.02
		Fitted	0.99	1.00	0.92	0.98	-4.62	-3.14
	MRTM2	Selected	1.00	1.00	0.99	0.99	-1.27	-1.49
		Fitted	0.99	1.00	0.99	1.00	-0.21	-0.17

Region 1 corresponds to the occipital cortex for the radioligand $[^{11}C]$AZ10419369 and the striatum for $[^{11}C]$SCH23390 Region 2 corresponds to the frontal cortex. Abbreviations: "SRTM" = simplified reference tissue model, "ref Logan" = reference tissue Logan plot, "MRTM2" = Ichise's Multilinear Reference Tissue Model 2 (MRTM2), "ICC" = intra-class correlation coefficient, "Pearson's r" = Pearson's correlation coefficient.

We also performed an analysis using PMOD weights and t* values in kinfitr for MA1 and MRTM2. We did not perform this analysis using the Logan methods, as these models were fitted using uniform weights in kinfitr (see Supplementary Materials S2). Additionally, 2TCM and SRTM were also run in kinfitr using the constant weights.

Agreement between kinfitr and PMOD using constant weights or kinfitr's default weighting scheme for the invasive models

			Pearson's r		ICC		Bias (%)	
**Ligand**	**Model**	**Weights**	**Region 1**	**Region 2**	**Region 1**	**Region 2**	**Bias 1**	**Bias 2**
[^{11}C]PBR28	MA1	Constant	1.00	0.99	0.97	0.97	8.60	10.21
		Default	1.00	1.00	0.95	0.97	10.53	10.06
	2TCM	Constant	1.00	0.99	0.98	0.97	8.43	12.17
		Default	1.00	1.00	0.99	1.00	2.01	1.19
[^{11}C]PK11195	MA1	Constant	1.00	1.00	1.00	1.00	0.08	0.09
		Default	0.99	0.95	0.97	0.89	5.07	9.63
	2TCM	Constant	1.00	0.98	1.00	0.98	1.23	0.59
		Default	1.00	0.98	1.00	0.98	1.16	0.69

Agreement between kinfitr and PMOD using constant weights or kinfitr’s default weighting scheme for the non-invasive models

			Pearson's r		ICC		Bias (%)	
**Ligand**	**Model**	**Weights**	**Region 1**	**Region 2**	**Region 1**	**Region 2**	**Bias 1**	**Bias 2**
[11C]AZ10419369	SRTM	Constant	1.00	1.00	1.00	1.00	0.33	0.25
		Default	1.00	1.00	1.00	1.00	-0.17	0.13
	MRTM2	Constant	0.95	0.94	0.78	0.77	-5.25	-5.74
		Default	0.97	0.96	0.87	0.81	-3.84	-5.24
[11C]SCH23390	SRTM	Constant	1.00	1.00	1.00	1.00	0.20	0.46
		Default	1.00	1.00	1.00	1.00	0.24	0.53
	MRTM2	Constant	0.99	1.00	0.99	0.99	-1.31	-1.37
		Default	1.00	1.00	0.99	0.99	-1.27	-1.49

Supplementary Materials S8

Relationship between Binding Outcomes between Models Estimated Using Each Tool

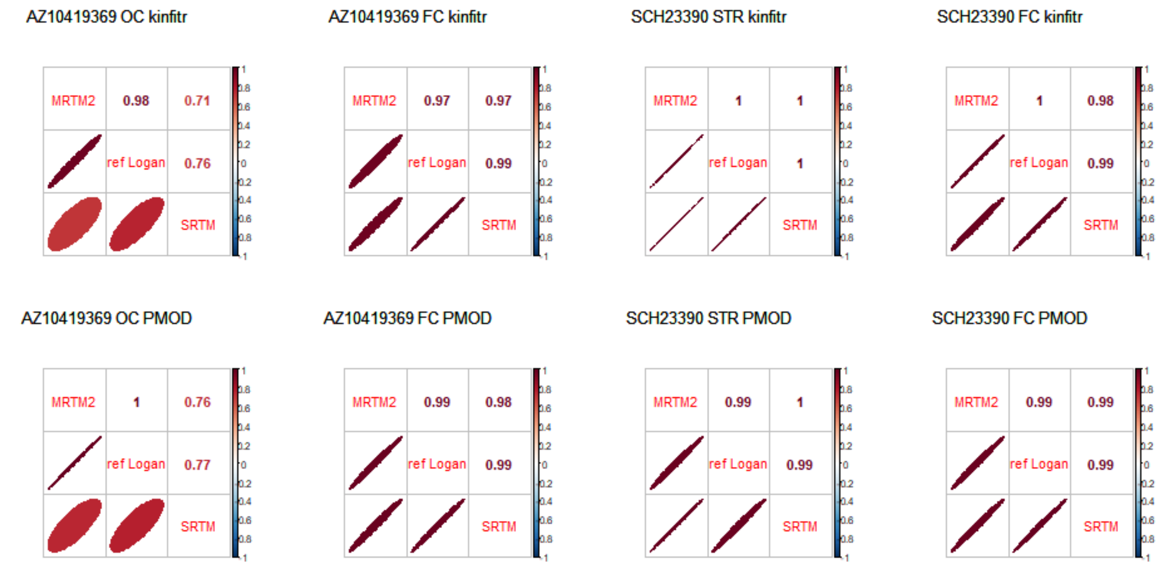

Correlations between the three non-invasive kinetic models for each region and radioligand for both kinfitr and PMOD. Abbreviations: OC = Occipital cortex, FC = Frontal cortex, STR = Striatum.

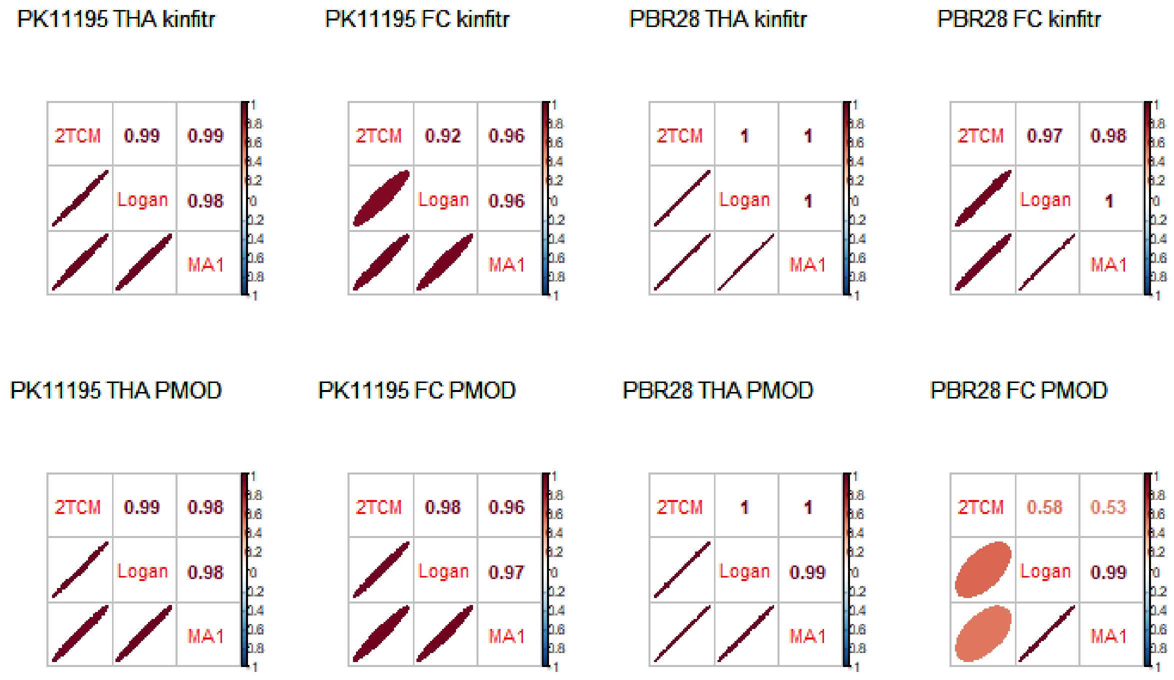

Correlations between the three invasive kinetic models for each region and radioligand for both kinfitr and PMOD. Abbreviations: THA = Thalamus, FC = Frontal cortex.

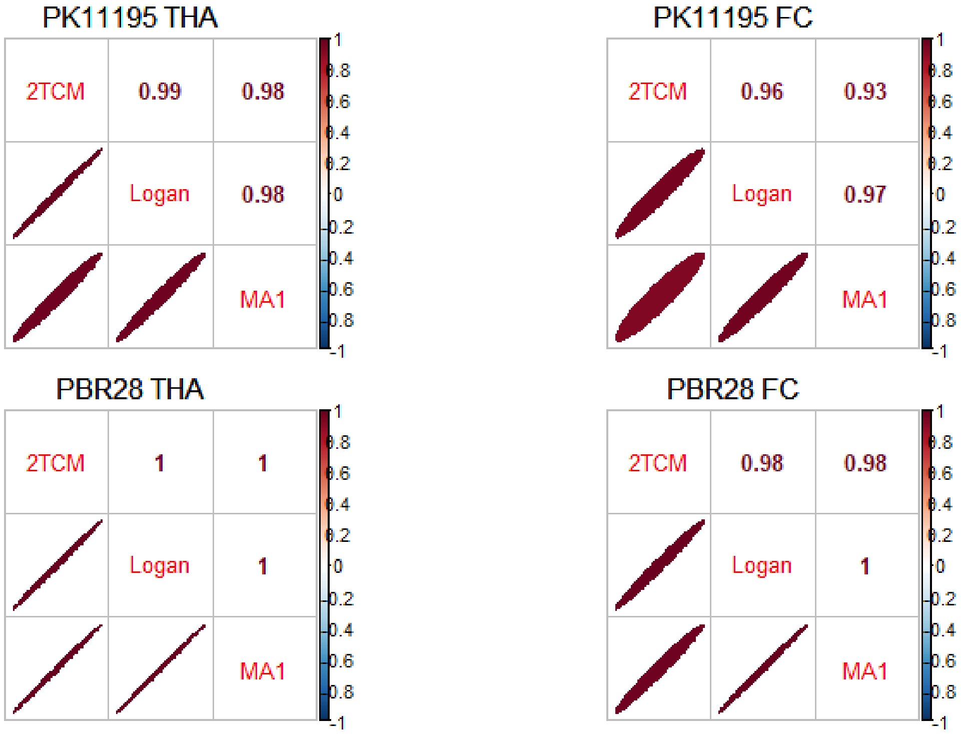

Correlations between the three invasive kinetic models for each region and radioligand for kinfitr using the t values which were fitted by PMOD. Abbreviations: THA = Thalamus, FC = Frontal cortex.*

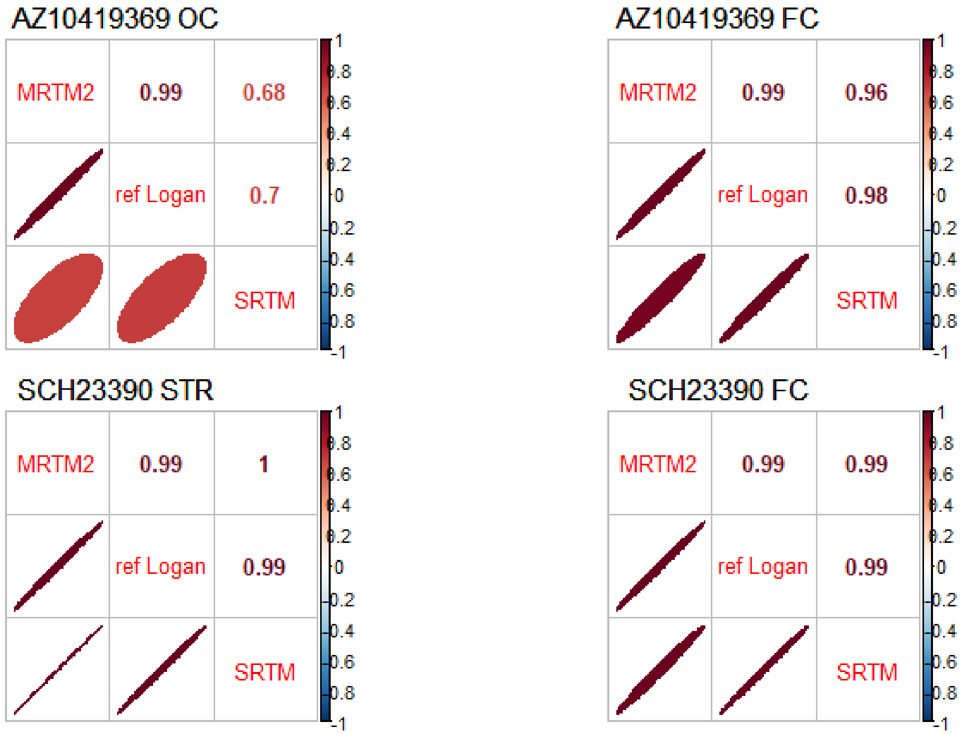

Correlations between the three non-invasive kinetic models for each region and radioligand for kinfitr using the t values which were fitted by PMOD. Abbreviations: STR = Striatum, OC = Occipital cortex, FC = Frontal cortex.*

Supplementary Materials S9

Test-retest analysis.

Test-retest reliability of kinfitr and PMOD for the invasive models using the higher-binding ROI

Ligand	Software	Model	Mean	CV (%)	ICC	WSCV (%)	AV (%)
[11C]PK11195	2TCM	kinfitr	0.76	27.1	0.75	13.9	18.9
		PMOD	0.75	27.4	0.72	15.0	20.0
	Logan	kinfitr	0.76	27.2	0.79	12.9	17.2
		PMOD	0.79	26.2	0.80	12.2	16.2
	MA1	kinfitr	0.84	26.4	0.73	14.1	17.9
		PMOD	0.80	28.2	0.67	16.6	19.2
[11C]PBR28	2TCM	kinfitr	3.84	59.6	0.91	18.4	25.1
		PMOD	3.73	57.3	0.89	19.1	26.7
	Logan	kinfitr	3.74	57.8	0.91	18.2	25.4
		PMOD	3.68	54.8	0.88	19.4	26.5
	MA1	kinfitr	3.99	58.7	0.91	18.4	24.7
		PMOD	3.54	53.1	0.91	16.7	23.7

Assessment of test-retest reliability of kinfitr and PMOD for a single high-binding ROI for the invasive models. The thalamus was used for both (R)- [11C]PK11195 and [11C]PBR28. Abbreviations: “2TCM” = Two-tissue compartmental model, “Logan” = Invasive Logan plot, “MA1” = Ichise’s Multilinear Analysis 1, “ICC” = intra-class correlation coefficient, “CV” = Coefficient of variance, “WSCV” = within-subject coefficient of variance, “AV” = absolute variability.

Test-retest reliability of kinfitr and PMOD for the non-invasive models using the higher-binding ROI

Ligand	Software	Model	Mean	CV (%)	ICC	WSCV (%)	AV (%)
[11C]AZ10419369	SRTM	kinfitr	1.59	10.7	0.67	6.3	5.9
		PMOD	1.60	11.0	0.67	6.5	5.9
	ref Logan	kinfitr	1.45	8.1	0.61	5.2	4.8
		PMOD	1.49	8.3	0.62	5.3	5.5
	MRTM2	kinfitr	1.42	8.2	0.52	5.8	4.7
		PMOD	1.48	8.0	0.59	5.2	5.5
[11C]SCH23390	SRTM	kinfitr	1.49	11.1	0.83	4.6	5.0
		PMOD	1.49	11.2	0.83	4.6	5.0
	ref Logan	kinfitr	1.48	11.3	0.82	4.8	5.3
		PMOD	1.56	12.3	0.80	5.6	7.0
	MRTM2	kinfitr	1.48	11.1	0.83	4.7	4.9
		PMOD	1.51	11.3	0.82	4.9	5.4

Assessment of test-retest reliability of kinfitr and PMOD for a single high-binding ROI for the non-invasive models. The occipital cortex region was used for the radioligand [11C]AZ10419369, the striatum for [11C]SCH23390. Abbreviations: “SRTM” = simplified reference tissue model, “ref Logan” = reference tissue Logan plot, “MRTM2” = Ichise's Multilinear Reference Tissue Model 2 (MRTM2), “ICC” = intra-class correlation coefficient, “CV” = Coefficient of variance, “WSCV” = within-subject coefficient of variance, “AV” = absolute variability.

Test-retest reliability of kinfitr and PMOD for the invasive models using the lower-binding ROI

Ligand	Software	Model	Mean	CV (%)	ICC	WSCV (%)	AV (%)
[11C]PK11195	2TCM	kinfitr	0.69	20.5	0.70	11.5	15.2
		PMOD	0.69	21.4	0.70	12.1	15.4
	Logan	kinfitr	0.67	22.6	0.73	12.2	15.2
		PMOD	0.71	21.5	0.77	10.7	12.5
	MA1	kinfitr	0.78	20.6	0.65	12.5	14.8
		PMOD	0.72	24.1	0.59	15.8	16.2
[11C]PBR28	2TCM	kinfitr	3.00	51.2	0.90	16.7	22.4
		PMOD	2.95	49.7	0.87	18.1	24.7
	Logan	kinfitr	2.91	53.1	0.91	16.0	22.2
		PMOD	2.88	50.0	0.89	16.6	23.0
	MA1	kinfitr	3.10	50.7	0.91	15.5	19.6
		PMOD	2.82	48.8	0.92	14.4	19.1

Assessment of test-retest reliability of kinfitr and PMOD for a single lower-binding ROI for the invasive models. The frontal cortex was used for both (R)- [11C]PK11195 and [11C]PBR28. Abbreviations: "2TCM" = Two-tissue compartmental model, "Logan" = Invasive Logan plot, "MA1" = Ichise's Multilinear Analysis 1, "ICC" = intra-class correlation coefficient, "CV" = Coefficient of variance, "WSCV" = within-subject coefficient of variance, "AV" = absolute variability.

Test-retest reliability of kinfitr and PMOD for the non-invasive models using the lower-binding ROI

Ligand	Software	Model	Mean	CV (%)	ICC	WSCV (%)	AV (%)
[11C]AZ10419369	SRTM	kinfitr	1.51	8.7	0.34	7.1	6.9
		PMOD	1.51	8.7	0.34	7.2	7.0
	ref Logan	kinfitr	1.46	8.7	0.33	7.2	7.4
		PMOD	1.52	8.8	0.32	7.3	7.4
	MRTM2	kinfitr	1.45	9.1	0.24	8.0	7.5
		PMOD	1.53	8.4	0.25	7.4	7.9
[11C]SCH23390	SRTM	kinfitr	0.29	17.4	0.51	12.3	13.7
		PMOD	0.29	17.5	0.51	12.3	13.7
	ref Logan	kinfitr	0.28	17.2	0.55	11.6	13.1
		PMOD	0.29	17.0	0.55	11.5	12.4
	MRTM2	kinfitr	0.27	17.3	0.58	11.3	13.0
		PMOD	0.28	16.9	0.59	10.9	12.2

Assessment of test-retest reliability of kinfitr and PMOD for a single lower-binding ROI for the non-invasive models. The occipital cortex region was used for the radioligand [^{11}C]AZ10419369, the striatum for [^{11}C]SCH23390. Abbreviations: "SRTM" = simplified reference tissue model, "ref Logan" = reference tissue Logan plot, "MRTM2" = Ichise's Multilinear Reference Tissue Model 2 (MRTM2), "ICC" = intra-class correlation coefficient, "CV" = Coefficient of variance, "WSCV" = within-subject coefficient of variance, "AV" = absolute variability.

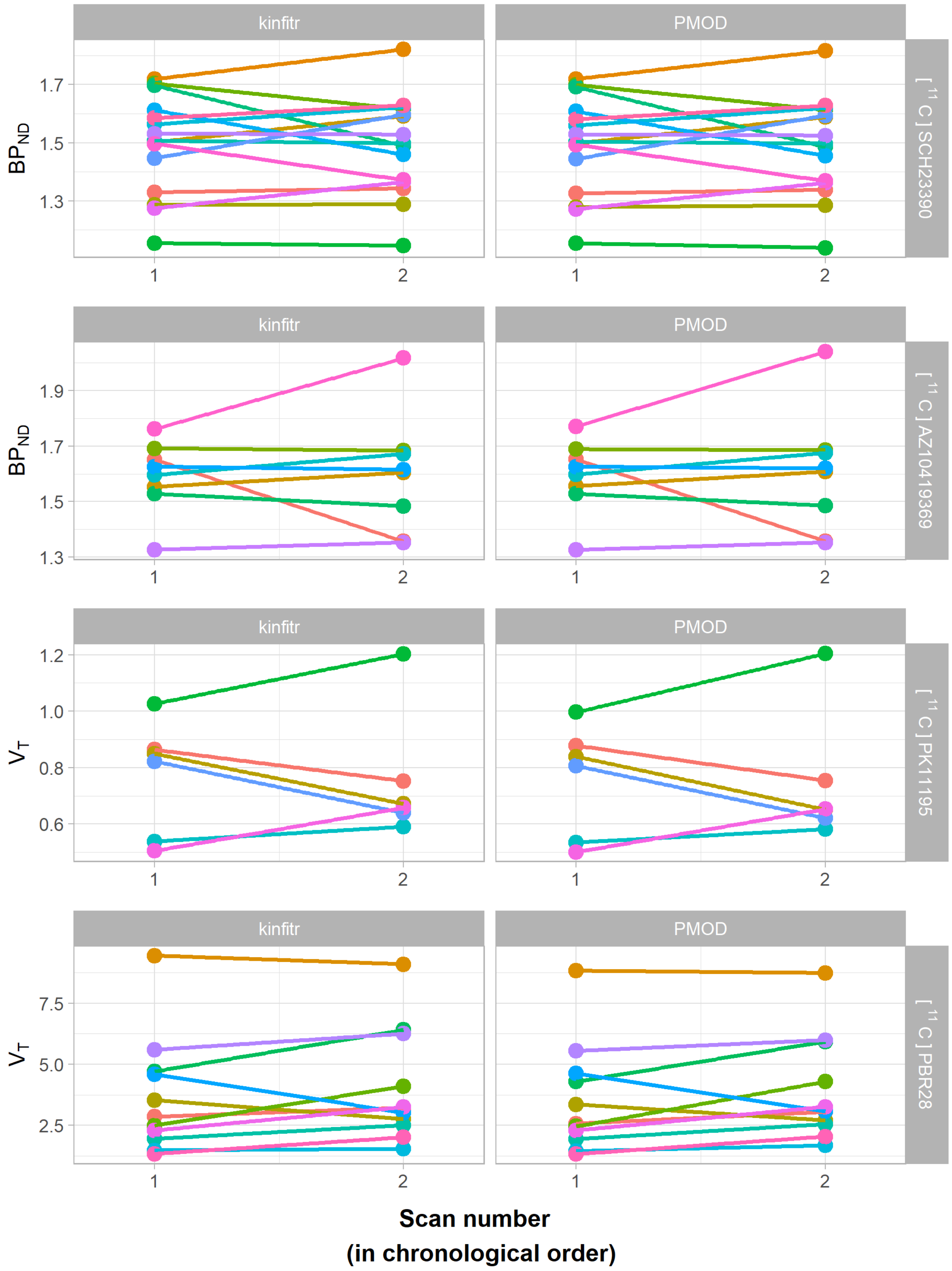

Binding estimate values comparing the first and second PET measurements. Each colour corresponds to a different individual, and the lines connect both of their two measurements. The ROIs used in making this figure were the higher-binding regions for each radioligand in this study, i.e. the occipital cortex for [^{11}C]AZ10419369, the striatum for [^{11}C]SCH23390 and the thalamus for both TSPO ligands. The kinetic models represented here are SRTM for the estimation of BP_{ND} (above two rows), and the invasive model 2TCM for the estimation of V_T

References

1. Audit Trail License – PMOD Technologies LLC [Internet]. [cited 2019 Jul 17]. Available from: https://www.pmod.com/web/?portfolio=41-audit-trail-license

2. PMOD Kinetic Modeling (PKIN) [Internet]. 1996 [cited 2019 Jul 17]. Available from: http://doc.pmod.com/PDF/PKIN.pdf

3. Xie Y (Mathematician), Allaire JJ, Grolemund G. R Markdown : the definitive guide. 303 p.
